# Depression and anxiety, an Indicated Prevention (DIP) protocol in homes for the elderly: feasibility and (cost) effectiveness of a stepped care programme

**DOI:** 10.1186/1471-2318-7-6

**Published:** 2007-03-08

**Authors:** Els Dozeman, Digna JF van Schaik, Aartjan TF Beekman, Wim AB Stalman, Judith E Bosmans, Harm WJ van Marwijk

**Affiliations:** 1 Department of General Practice, VU University Medical Centre, Amsterdam, The Netherlands; 2 Institute for Research in Extramurel Medicine, VU University Medical Centre, Amsterdam, The Netherlands; 3 Department of Psychiatry, VU University Medical Centre, Amsterdam, The Netherlands; 4 Institute for Health Sciences, Faculty of Earth and Life Sciences, VU University, Amsterdam, The Netherlands

## Abstract

**Background:**

Depressive and anxiety disorders are a very common, serious and underdetected problem in homes for the elderly. Elderly persons in residential homes are at high risk for developing major depressive and anxiety disorders, and, therefore, deserve attention with regard to prevention.

**Methods/Design:**

This protocol describes a randomised trial on the feasibility and (cost) effectiveness of a stepped-care programme for prevention of depressive and anxiety disorders in homes for the elderly. The main outcome measure is the incidence of depressive and anxiety disorder in one year with a two years follow up. Secondary outcomes are symptoms of depression and anxiety, quality of life, direct health care costs and satisfaction with treatment.

**Discussion:**

The number of studies examining the effects of preventive interventions on the incidence of mental disorders in the elderly population is very small. However, indicated prevention by means of a stepped-care programme seems to be an important option for decreasing the burden of illness for residents and their caregivers. This study contributes to the body of knowledge in this field. Positive effects may contribute to further use and development of tailored, (cost-) effective and easy to use interventions in a preventive stepped-care programme.

**Trial Registration:**

The Dutch Cochrane Centre, ISRCTN27540731

## Background

In homes for the elderly, depressive and anxiety disorders are very common and have a large impact on the well-being and daily functioning of the residents. In 2002 over 90,000 of the nearly one million elderly persons in the Netherlands, aged 75 years or older, lived in a residential home [1]. Up to 30 percent of these residents develop symptoms of depression and anxiety, such as apathy and feelings of loneliness and hopelessness [2]. In about 30 to 35 percent of residents with symptoms of depression and anxiety, these symptoms eventually develop into major depression and generalised anxiety disorder [3,4]. These disorders are often associated with a poor prognosis and with excess mortality, disability, handicap and service utilisation. [5-11]. Treatment can reduce the illness burden in the population, but only to a moderate extent [12]. Therefore, prevention may be an attractive option.

Indicated prevention, as opposed to universal prevention (aimed at the general population regardless of risk status) or selective prevention (aimed at high-risk groups), aims to counter the onset or development of a disorder in those who already have symptoms. Indicated prevention may be a successful approach since it targets high-risk persons who are identified as having minimal symptoms foreshadowing mental disorders. Risk factors for depression and anxiety disorders in the general population are known; a large naturalistic follow-up study among persons 55 years of age in the community showed that a set of six indicators explained 83% of the total prognostic variance. The prediction model include symptoms of depression and/or anxiety disorder, functional limitations, small network, female gender, low education or suffering from chronic diseases [13]. These characteristics are very common among residents of homes for the elderly, which makes this a population at high risk for depressive and anxiety disorders. Indicated prevention studies, in different target populations, have shown that several interventions are capable of reducing the incidence of depression and anxiety disorder up to approximately 30% [14]. From an ethical and societal point of view, indicated prevention is preferred to universal prevention and selective prevention. Prevention activities may increase fear or uneasiness in the target population; however, this is legitimated most for people who already suffer of symptoms and are known to have a high risk.

Although caregivers in the homes recognize the impact of the problem as described earlier, symptoms of depression and anxiety are rarely identified and labelled as such [15,16]. A substantial part of residents of homes for the elderly suffers from physical co-morbidity. Symptoms of depression and anxiety disorder like apathy and weariness may also be ascribed to physical illnesses [17]. This makes that symptoms of anxiety and depression are often interpreted only as consequences of decline rather than problems that require intervention. Physical illness in the elderly is most of the time progressive and hard to cure. Depressive and anxiety disorders, however, have more optimistic possibilities for prevention and treatment [14,18,19]. Moreover, as the population is ageing rapidly and the policy in the Netherlands is to facilitate independent living as much as possible, the level of psychological and physical frailty in homes for the elderly has been increasing rapidly. At the same time, medical care is also changing: General Practitioners (GPs), who are responsible for medical care in the residential homes in the Netherlands, have for instance halved the number of house visits since 1987 [20]. In addition, most homes suffer from staff deficits, in which cases the quality of care is threatened [21]. Therefore, efficient use of the available, scarce resources, as supplied by this stepped care protocol, is of great importance.

This study focuses on the feasibility and (cost-) effectiveness of a stepped-care intervention to prevent the onset of full-blown depressive and anxiety disorders among residents of homes for the elderly with subthreshold anxiety and/or depressive symptoms. The stepped-care protocol chosen in this study is at the moment already being used in a related study for vulnerable elderly persons in the community [22]. This program is based on the assumption that stepping up from lower, less intensive to higher, more intensive levels of preventive activities, based on monitoring outcomes, may increase the (cost) effectiveness of the programme and maximize efficiency of resource allocation [23-25]. Moreover, it is reasonable to assume that different people prefer and require different levels of preventive activities. Some may be helped with working through a self-help manual, others could benefit from a minimal-contact psychotherapy, and still others may require a form of pharmacotherapy or more intensive individual psychological treatment. In this study we focus on residents with subsyndromal symptoms of depression and/or anxiety who do not meet the criteria of a full-blown disorder. A stepped-care protocol may be particularly relevant for this group. Residents that already suffer from disorders might be better managed through more complex and intensive collaborative-care models [24,26]. The primary aim of the present trial is to investigate whether a stepped-care protocol based on monitoring and evidence-based interventions is able to prevent the onset of depressive and anxiety disorders in residents of homes for the elderly as compared to care as usual. Secondary aims include reducing the level of symptoms of depression and anxiety, improving quality of life, reducing costs of health care consumption and improving satisfaction with treatment. The feasibility of this protocol is judged on the basis of motivation and evaluation of residents, caring staff and general practitioners. Organisational barriers are also assessed.

## Methods/Design

### Design

To evaluate the effects of the stepped care protocol, a randomised, controlled trial in residential homes for the elderly will be carried out. Homes for the elderly in Amsterdam will be selected. Caring staff and GPs of the participating residents will be involved in the intervention protocol. Participants are asked to give informed consent referring to the stepped care interventions, the randomisation and informing their GP during the intervention programme. The medical Ethics Committee of the VU medical centre in Amsterdam approved the study design.

### Participants

Participants will be recruited among residents of homes for the elderly by means of a screening procedure using the Centre of Epidemiologic Studies Depression scale (CES-D 20) as a screening instrument [27-29]. Residents with a score of 16 and more are invited for a follow-up diagnostic interview. This score is widely accepted as an indication for symptoms of depression and/or anxiety [30]. Residents meeting criteria for major depression and/or clinical anxiety disorder according to the Mini International Neuropsychiatric Interview (MINI) [31,32] or suffering cognitive impairment according to the Mini Mental State Examination (MMSE <21) are excluded. Residents capable of informed consent and with sufficient understanding of the Dutch language, are eligible for the study.

### Interventions

#### Stepped-care protocol

Our intervention protocol is based on the protocol of The Prevention of Anxiety and Depression in Late Life consortium (PADLL, in which several Dutch universities and the national mental health institute collaborate) [22]. This consortium designed a generic stepped-care intervention protocol to be tested across different health care settings, different groups of elderly persons and different forms of prevention (prevention of episodes vs. relapse prevention). For use in homes for the elderly, we selected the interventions that seemed to be most suitable for this frail and isolated population. In organizing this stepped-care approach, evidence-based guidelines are used as far as possible. Staff in the homes for the elderly and mental health nurses will be contracted to cooperate in the stepped-care protocol. One caregiver will coordinate the care. Necessary training will be provided. When residents show continuously elevated CES-D scores, GPs of the residents will be involved.

**Figure 1:**
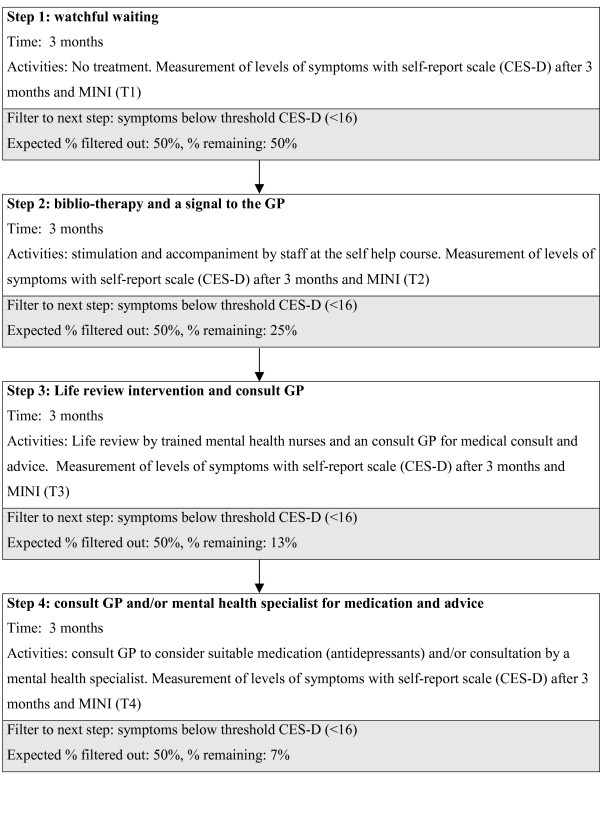
stepped care protocol.

The protocol contains the following steps (see Figure 1):

##### Step 1: watchful waiting

The first step of the program consists of watchful waiting, since frequently (up to 50% of the cases) the complaints disappear without active intervention [33]. Residents will be included in the intervention group in step 2 after a three months period of persisting symptoms.

##### Step 2: biblio-therapy and a signal to the GP

Participants, who still have symptoms of depression and anxiety after 3 months of watchful waiting, as measured with the CES-D, are informed about the possibility of learning to cope with these symptoms by a self-help course for coping with depression and anxiety with an accent on activity scheduling. Self administered treatments for depression and anxiety have proven to be successful in clinical practice and for older adults [19,34-39]. Staff will be trained to accompany and stimulate the residents while working through the course in their own tempo. At the same time, GPs will be involved by informing them about the persisting symptoms. After a period of three months the third measurement will take place.

##### Step 3: Life review intervention and consult GP

When symptoms persist, residents are informed that they qualify for a more specific treatment: the life review with problem solving accents. Life review is a promising treatment for elderly with symptoms of depression [40]. The effect for elderly with symptoms of anxiety is not explicitly studied, however support exists for a decrease in disempowerment themes, such as anxiety and despair [41]. The intervention will be delivered by a mental health nurse trained and supervised in the intervention. This training will be given by experienced supervisors of the national mental health institute (Trimbos institute), who adapted this intervention for the use in homes for the elderly in the Netherlands. GP's of participants in step 3 receive advise to check for possible somatic causes of depression and anxiety symptoms (thyroid disease, vitamin deficiencies, Parkinson disease) or intake of depressogene substances (medication, alcohol). After a period of three months the fourth measurement will take place.

##### Step 4: consultation mental health specialist

In case the residents still have CES-D scores > 15, they will receive advice to consult their GP to consider medication (antidepressants) and/or consultation by a mental health specialist.

#### Usual care

In the Netherlands, residential homes provide daily care to the infirm elderly with significant limitations to daily living; if needed, they also provide basic medical care. Nurses and care workers supply generic care, while GPs are medically responsible. Residents assigned to the control group receive care as usual by staff of the homes and their GPs. Residents are informed that every three months measurements with CES-D and additional interviews take place in order to keep track of the symptoms.

### Data collection

#### Primary outcome

##### Depressive/anxiety disorder

Both the intervention and control participants will receive blinded independent assessment interviews of depression and anxiety status with the Mini-International Neuropsychiatric Interview (M.I.N.I.), at baseline, 3, 6, 9, 12 and 24 months (see Table 1). The M.I.N.I. is a short structured diagnostic interview for DSM-IV and ICD-10 psychiatric disorders. With an administration time of approximately 15 minutes, it was designed to meet the need for a short but accurate structured psychiatric interview for multicenter clinical trials and epidemiology studies and to be used in outcome tracking in non research clinical settings [32].

#### Secondary outcome

##### Depressive symptoms

Depressive symptoms are monitored by means of the Centre for Epidemiologic Studies Depression Scale CES-D. This instrument is designed specifically for the screening of depression but has also been found to be a satisfactory screener for anxiety disorders [42]. It consists of 20 items and its total score has a range between 0 and 60. Scores > 15 indicate clinical significant levels of depressive and anxiety symptoms. The CES/D is also used for follow up purposes.

##### Anxiety symptoms

For follow up measurements of symptoms of anxiety the seven anxiety items of the Hospital Anxiety and Depression scale (HADS-A) [43] will be used in addition to the CES-D. The aim is to optimise the sensitivity and specificity of the CES-D for subthreshold anxiety.

##### Quality of life

Quality of life will be measured using the EuroQol. Using the British and the Dutch tariff [44,45], the 5 dimensions of the EuroQol will be valued to obtain utilities. Subsequently, Quality Adjusted Life Years (QALYs) will be calculated by multiplying the utilities with the amount of time a resident spent in this health state.

##### Health care utilization

A cost-effectiveness analysis will be performed from a societal perspective. Direct health care costs (e.g. visit to GP, specialist and use of medicines) and direct non-healthcare costs (self medication) are assessed with an interview based on the Trimbos/iMTA questionnaire for Costs associated with Psychiatric Illness (TiC-P). This questionnaire is developed by the Trimbos Institute Utrecht in combination with the institute for Medical Technology Assessment Rotterdam. Since all patients were living in a home for the elderly lost productivity costs and costs of informal and formal care are not relevant in this study. Resource use was measured using an interview that was based on the TiC-P. All direct costs are considered because it is difficult to determine which costs are associated with the depression and which are not. Resource use is valued using Dutch standard costs [46]. Medication costs will be valued using prices of the Royal Dutch Society for Pharmacy [47].

##### Patient satisfaction

To measure satisfaction with the treatment, the "GGZ thermometer, developed by the national mental health institute (Trimbos), is chosen. The instrument focuses on the appreciation of treatment explanation, the caring staff and the result of the interventions.

#### Effect modifiers

We assess the following potential effect modifiers on the level of de residents: presence of chronic illnesses and handicaps [48], social support [49], loneliness [50], personality (mastery) [51] and socio demographic characteristics, such as age, gender, level of education and marital status.

Table 2 displays the outcome parameters and instruments and additionally used questionnaires

### Sample size

Based on the prospective data of the Longitudinal Ageing Study Amsterdam (LASA) we conservatively estimate the incidence rate of depressive and/or anxiety disorder of elderly with symptoms of anxiety and/or depression within two years at 35% [33,52]. Our aim is to reduce incidence of full blown anxiety and depression disorders in the prevention group to 20%. To be able to detect this reduction (alpha = 0.5 and power = 0.80) 134 completers are needed, 67 in both conditions [53]. In the population of elderly persons in residential homes, substantial drop out (attrition) is expected because of illness and mortality. This is estimated at 20%, so therefore we need 172 participants. We know from previous projects that two thirds of subjects complete the CES-D, and we assume 40% will score 16 or more [22]. We estimate that 20% of these subjects already have a depressive and/or anxiety disorder and therefore will be excluded. With 80% consenting for randomization, we conservatively need to screen 1200 residents to find 86 residents per arm.

### Randomization

At least 172 residents will be included; 86 will start in the intervention group who follow the stepped care protocol. The other 86 will receive care as usual. Subjects are randomized at the patient level per home in computer generated blocks of four by an independent statistician, simultaneously with the inclusion in the study. Blocking is used to ensure that comparison groups are of approximately the same size per home.

### Blinding

Interviewers are kept blind from the randomization status of participants. It is not possible to keep the residents blinded. Staff members in the homes for the elderly and GPs are not informed about allocation of the residents. However, as residents receive questionnaires, staff will be aware of participation of the residents in the project.

### Analysis

To examine differences on the primary outcome, survival analysis will be used. The secondary outcome measures are analysed using random coefficient analyses. The analyses will be performed according to the "intention to treat" principle. Additionally, data will be analyzed according to the per protocol principle. Differences in total costs between both groups are assessed and confidence intervals are estimated using bias-corrected and accelerated bootstrapping with 2000 replications [54]. Cost-effectiveness and cost-utility ratios will be calculated by dividing the difference in total costs between the intervention and usual care group by the difference in the clinical effect measures and QALYs between the treatment groups. Uncertainty around the cost-effectiveness and cost-utility ratios will calculated using the bias-corrected percentile bootstrapping method (5000 replications) [55]. The bootstrapped cost-effect pairs were plotted on a cost-effectiveness plane.

## Discussion

By presenting the design of this study before the results are available we aim to offer researchers the opportunity to consider the methodological quality of this study with a critical view. Caregivers can benefit by considering the information on the practical applications of the proposed protocol on a vulnerable group of residents of homes for the elderly.

The importance of psychiatric disorders to public health makes prevention a serious issue. Indicated prevention has proven to be a very promising field [56]. Nevertheless, the number of studies examining the effects of preventive interventions on the incidence of mental disorders is very small, especially among elderly [14]. With this study we aim to supply more scientific knowledge to base guidelines for this kind of interventions on.

This study evaluates the effectiveness of a stepped care protocol for prevention of depression and anxiety in homes for the elderly in the Netherlands, while a similar study is conducted in the community. This offers the possibility to compare the results in these different elderly populations. This comparison may supply further information about effectiveness and feasibility of this prevention strategy in different populations of the elderly.

Another strength of the chosen prevention strategy is the focus on the combination of symptoms of depression and anxiety. Risk factors for the onset of these disorders overlap [57,58]. Focussing on the combination of symptoms of a target group with high risk, may therefore increase the effect of the intervention.

The randomization is at the patient level. The risk of confounding is merely applied to the one step in the intervention protocol where staff members, all or not provide and stimulate the self-help manuals. Therefore extra efforts will be attended to instructing the staff. Moreover, research on training and education of caregivers in primary care [59-61] shows that the influence of short courses on attitude in the management of patients with mental illness as on patient outcomes is relatively small. Therefore, contamination with the usual care group is neglectable. Finally, the main disadvantage of cluster randomisation, either on ward or on home level, is lack in unity of wards and homes.

The use of a self-help course as a second step in the protocol has numerous advantages. In the first place, it can be an efficient and high quality form of therapy [35]. Secondly, a self-help course may lower the threshold for those who don't want or dare to ask for therapist treatment. Moreover, it may contribute to the effectiveness of the following step (if necessary) by preparing the client for therapeutic treatment. On the other hand, unsuccessfully treatment may cause less willingness to undergo further, more involved treatment. Therefore, a thorough explanation of the rationale and procedures of the stepped care model is important.

Activities in this prevention program are addressed to people who are at risk to develop a serious disorder, but have no explicit request for treatment. The interventions therefore need to be non threatening and short time limited. Life review seems to be a very useful method as it is a non-stigmatising, easy to use and easily administered treatment method, suitable for elderly residents [40,62].

The results of this RCT will provide valuable information about the feasibility and (cost)effectiveness of a stepped care protocol for prevention of depression and anxiety disorders in residential homes for the elderly. Moreover, positive effects may stimulate the further development and use of guidelines by caregivers in homes for the elderly for psychiatric issues and thereby improve the quality of living in these environments. The start of the study is anticipated for October 2006 with results available in 2010.

## Competing interests

The author(s) declare that they have no competing interests.

## Authors' contributions

HvM and DvS developed the design of the randomized clinical trial and participated in writing the article. WS and AB advised on the content of the article and supervise the project. JB performs the cost-effectiveness analysis and advised on the content of the article. ED is the principal investigator and writer of this manuscript. All authors have read and approved the final version of the manuscript

## Pre-publication history

The pre-publication history for this paper can be accessed here:



## Figures and Tables

**Table 1:** time table for data collection

		T0	T0-a	T0-b	T1 3 mnd	T2 6 mnd	T3 9 mnd	T4 12 mnd	T5 24 mnd
consent		x							
CES-D	x				x	x	x	x	x
M.I.N.I.			x		x	x	x	x	x
MMSE			x					x	x
Socio demographic characteristics				x					
HADS-A				x	x	x	x	x	x
Hearing and seeing				x				x	x
Chronic illness and handicap (GARS)				x				x	x
History of illness and life events (DIS)				x				x	x
Social support				x				x	x
Personality (Mastery)				x				x	x
TIC-P				x	x	x	x	x	x
Euroqol				x	x	x	x	x	x
Pt. satisfaction						x	x	x	

**Table 2:** Instruments used to establish primary and secondary outcomes

**Outcome measures**	**Instruments**
**Primary outcome**	
Depressive/anxiety disorder	MINI diagnostic interview
**Secondary outcome**	
depressive symptoms	CES-D
anxiety symptoms	HADS-A
Quality of life	Euroqol
Health care utilisation	TiC-P
Patient satisfaction	GGZ thermometer
**Effect modifiers**	
Chronic illness and handicap	Medical file and GARSscale
Social support	Social support list
Personality	Mastery scale
Loneliness	De Jong-Giersveld
Socio demographic characteristics	
